# Cardiac progenitor cell therapy: mechanisms of action

**DOI:** 10.1186/s13578-024-01211-x

**Published:** 2024-03-05

**Authors:** Rut Bryl, Magdalena Kulus, Artur Bryja, Dominika Domagała, Paul Mozdziak, Paweł Antosik, Dorota Bukowska, Maciej Zabel, Piotr Dzięgiel, Bartosz Kempisty

**Affiliations:** 1 Section of Regenerative Medicine and Cancer Research, Natural Sciences Club, Faculty of Biology, Adam Mickiewicz University, Poznań, Poznan, 61-614 Poland; 2 Department of Veterinary Surgery, Institute of Veterinary Medicine, Nicolaus Copernicus University, Torun, 87-100 Poland; 3 Department of Human Morphology and Embryology, Division of Anatomy, Wroclaw Medical University, Wroclaw, 50-367 Poland; 4 Prestage Department of Poultry Science, North Carolina State University, Raleigh, NC 27695 USA; 5 Physiology Graduate Faculty, North Carolina State University, Raleigh, NC 27695 USA; 6 Department of Diagnostics and Clinical Sciences, Institute of Veterinary Medicine, Nicolaus Copernicus University in Torun, Torun, 87-100 Poland; 7 Division of Anatomy and Histology, University of Zielona Góra, Zielona Góra, 65-046 Poland; 8 Department of Human Morphology and Embryology, Division of Histology and Embryology, Wroclaw Medical University, Wroclaw, 50-368 Poland; 9 Department of Obstetrics and Gynaecology, University Hospital and Masaryk University, Brno, 62500 Czech Republic

**Keywords:** Cardiac regeneration, Adult cardiac progenitor cells, Exosomes, Non-coding RNAs

## Abstract

Heart failure (HF) is an end-stage of many cardiac diseases and one of the main causes of death worldwide. The current management of this disease remains suboptimal. The adult mammalian heart was considered a post-mitotic organ. However, several reports suggest that it may possess modest regenerative potential. Adult cardiac progenitor cells (CPCs), the main players in the cardiac regeneration, constitute, as it may seem, a heterogenous group of cells, which remain quiescent in physiological conditions and become activated after an injury, contributing to cardiomyocytes renewal. They can mediate their beneficial effects through direct differentiation into cardiac cells and activation of resident stem cells but majorly do so through paracrine release of factors. CPCs can secrete cytokines, chemokines, and growth factors as well as exosomes, rich in proteins, lipids and non-coding RNAs, such as miRNAs and YRNAs, which contribute to reparation of myocardium by promoting angiogenesis, cardioprotection, cardiomyogenesis, anti-fibrotic activity, and by immune modulation. Preclinical studies assessing cardiac progenitor cells and cardiac progenitor cells-derived exosomes on damaged myocardium show that administration of cardiac progenitor cells-derived exosomes can mimic effects of cell transplantation. Exosomes may become new promising therapeutic strategy for heart regeneration nevertheless there are still several limitations as to their use in the clinic. Key questions regarding their dosage, safety, specificity, pharmacokinetics, pharmacodynamics and route of administration remain outstanding. There are still gaps in the knowledge on basic biology of exosomes and filling them will bring as closer to translation into clinic.

## Heart failure as a worldwide problem and heart regenerative potential

Advances in cardiovascular medicine and surgery in the past three decades have led to substantial decrease in mortality associated with acute cardiovascular syndromes in developed countries. Unfortunately, in many cases the occurrence of myocardial damage eventually leads to development of heart failure [1].

Heart failure (HF) is a chronic phase of many cardiac diseases, including coronary artery disease (and cardiomyopathies. According to AHA and ACC Foundation HF is “a complex clinical syndrome that results from any structural or functional impairment of ventricular filling or ejection of blood” [2]. It is a global epidemic, with a prevalence of approximately 37.7 million [3]. Increasingly, HF occurs in middle- and low-income countries, where lifestyle favors occurrence of HF risk factors – development of obesity, diabetes and hypertension [1]. HF remains a substantial burden to the health care system and is one of the major causes of hospitalization, especially in the elderly [4, 5]. Average survival of patients with HF diagnosis varies from 3 to 5 years, thus prognosis of this disease is poorer than for most cancers [1, 6]. The total cost associated with HF in 2020 in the USA was approximately $43.6 billion, of which the vast majority were medical costs [7].

Certain drugs including β-adrenoreceptor blockers, angiotensin-converting enzyme inhibitors, mineralocorticoid receptor antagonists, angiotensin receptor-neprilysin inhibitor and sodium-glucose co-transporter 2 inhibitors have been used as well as devices for cardiac rhythm management including implantable cardioverter-defibrillators and cardiac resynchronization therapy have been considered as management options [8]. In advanced heart failure there are 2 main treatment strategies - heart transplantation and use of ventricular assisted devices [9]. Nevertheless, the results of treatment remain mixed, and the management mostly improved the survival of patients with chronic heart failure with reduced ejection fraction [10]. Heart transplantation remains a method of choice for end-stage heart failure, but it possesses several limitations, of which the major one is a chronic shortage of donors [9]. Due to the growing number of cardiovascular patients and the continuing shortage of donors, the use of artificial hearts is gaining popularity. A total artificial heart (TAH) is a type of pneumatic mechanism that is inserted to replace the non-functioning native parts of the organ. It is used in end-stage heart failure, especially in patients waiting for a transplant. The most widely used device is the SynCardia TAH, which has been used in more than 2,000 patients, with 1-year survival remaining at 42%. Survival rates increase significantly for patients who have survived to the target heart transplant [11–13].

New, optimal methods of treating heart failure are necessary. The adult mammalian heart was long considered a post-mitotic organ, however, there were recently several reports which suggest that it may possess some modest intrinsic regenerative potential. Strategies employing cells responsible for heart regeneration may become a promising alternative to current disease management.

The heart has been long considered an organ unable to regenerate and renew. The majority constitute of cardiomyocytes, which are terminally differentiated and undividable. They function through the whole lifetime and show high resistance to death. The only response to aging and loss of contractility is hypertrophy by increase in size to accommodate a larger number of sarcomeres [14, 15]. Recent studies suggest that new cardiomyocytes are generated in response to exploitation/physiological wear and tear, and particularly injuries [16–19]. This turnover is specific to mammals’ species and its quantification remains problematic, mostly due to limitation of methodology [20]. Based on radioactive isotope decay, Bergman et al. have reported that the annual turnover of cardiomyocytes amounts to 1% for 20-year-olds and successively decreases to 0.3% for 75-year-olds [16, 21]. As to rodents, Senyo et al. have shown that adult murine hearts cell turnover is approximately ~ 1% per year and can be accounted exclusively to division of pre-existing cardiomyocytes [22]. In other studies, these values vary from 0 to 4% annually [19, 23]. Although it remains certain that the myocardial regenerative response is not able to counterbalance CM loss and injury, there is some possibility that the cell turnover occurs due to presence of endogenous pool of cardiac progenitor cells (CPCs) [24].

## Resident cardiac progenitor cells

Cardiac progenitor cells are a specific type of stem cells found in adult heart tissues. A characteristic feature is the expression of specific makers like receptor tyrosine kinase, c-Kit and other. The discovery of CPCs has proven the repair capabilities of heart tissue [25]. Their properties contribute to the regeneration of tissue after an injury such as a heart infarction. It is suggested that CPCs promote cardiomyocyte proliferation, angiogenesis, and increase blood flow, leading to regeneration after injury. In addition, it is suggested that they inhibit apoptosis, reduce fibrosis and inflammation, resulting in a smaller scar [26]. Due to their stem-like properties, they can differentiate into various cell types, including cardiomyocytes, endothelial and smooth muscle cells. However, the regenerative capacity of cardiac tissue is limited due to the small number of CPCs, large areas of extensive damage or the immaturity of the cardiomyocytes formed [27]. Currently, based on a range of ongoing studies, it is suggested that the mechanism of action of CPCs is mainly through local protection of endogenous tissues rather than direct differentiation [28]. Cardiac progenitor cells constitute, as it may seem, a heterogenous group of cells, which remain quiescent in physiological conditions and become activated after an injury, contributing to CM renewal [17]. These cells can be localized in various regions of the heart (atria, ventricles, epicardium or pericardium) [29]. Several studies aimed at identification and isolation of endogenous pool of such cells in the adult hearts of mammals such as mouse, rat, pig, or eventually human. The main subpopulations are presented in the Table 1. Such cells should be characterized by clonogenicity, self-renewal, differentiation into several cell types of cardiac lineage, including cardiomyocytes, vascular smooth muscle cells and endothelial cells (ECs) in vitro and in vivo, expression of transcription factors (Isl-1, Nkx2.5, MEF2C, and GATA-4) and several stemness markers (Oct3/4, Bmi-1, and Nanog). In some cases, cardiosphere formation is also tested. In preclinical myocardial infarction (MI) models, intra-myocardial transplantation of cardiac progenitor cells leads to reduction of myocardial scar and, in some cases, preservation of left ventricular function: [24, 29, 30].

**Table 1:** Summary of cardiac progenitor cells populations. Adapted from [24, 29, 31]

Cell type (or phenotype)	Markers used for isolation and characterization	Source
Cardiac colony-forming unit fibroblasts (cCFU-Fs)	Sca-1^pos^, PDGFR-α^pos^, CD31^neg^, c-Kit^low^, CD45^neg^, FLK1^neg^, CD44^pos^, CD90^pos^, CD29^pos^ and CD105^pos^	mouse, human
Cardiac side population cells (CSPCs)	CD34^pos^, CD45^pos^, Abcg2^pos^, Sca-1^pos^, c-kit^pos^, NKX2–5^neg^, GATA-4^neg^	mouse, human
Cardiosphere-derived cells (CDCs)	CD31^pos^, CD105^pos^, CD34^pos^, CD45^pos^, Abcg2^pos^, Sca1^pos^, c-kit^low^	mouse, rat, dog, pig, human
c-kit^pos^ eCSCs	CD34^neg^, CD45^neg^, Sca-1^pos^, Abcg2^pos^, CD105^pos^, CD166^pos^, GATA-4^pos^, NKX2–5^pos/neg or low^, MEF2C^pos^, VEGFR-2^neg^, CD31^neg^,	mouse, rat, pig, human
Epicardium-derived progenitor cells (EPDCs)	CD34^pos^, c-Kit^pos/neg^, CD44^pos^, CD90^pos^, CD105^pos^, CD46^pos^, WT-1	mouse, human
Isl1^pos^ CPCs (embryonic/fetal)	CD31^neg^, Sca-1^neg^, c-kit^neg^, GATA-4^pos^, NKX2–5^pos^	mouse, rat, human
Sca1^pos^ CPCs	Sca-1^pos^, CD105^pos^, CD34^neg^, CD45^neg^, FLK1^neg^, c-kit^pos/neg^, GATA-4^pos^, NKX2-5^pos/neg^, MEF2C^pos^; CD133^neg^	mouse, human

The two well characterized cardiac progenitor cell populations include c-kit^pos^ cells and cardiosphere derived cells (CDCs). The use of these cell populations in preclinical studies is shown in Table 2.

A number of preclinical studies have been conducted on small animals. This model is economically justified and enables relatively quick experiments that can be statistically evaluated. However, it should be kept in mind that positive results in small animals may not always be replicated in the same manner in clinical trials.

In 2003, CPCs were characterized, termed Lin^−^ c-kit^POS^ which exhibit c-kit expression but are negative for typical hematopoietic lineage markers. These cells were clonogenic, self-renewing and multipotent. Upon injection of c-kit^pos^ cells to the rat hearts after MI, reconstitution of myocardium was observed [25]. Simultaneously, based on the conducted research, c-kit^POS^ cells have been identified in the adult hearts of several mammals [31–33]. However, a larger population of these cells was observed at birth than in adult animals [33]. It should be noted that injection of c-kit^POS^ cells in the post-infarction area was associated with an increase in the population of these cells in cardiac tissue [32]. Greater interest in c-kit^POS^ cells and their positive effect on the reduction of post-infarction scar [25, 32] contributed to the development of a protocol for the isolation of c-kit^POS^ cells from the hearts of mice and rats [31]. In addition, it was observed that c-kit^POS^ cells with high GATA-4 expression significantly affected cardiomyocyte viability [33]. Similar properties were observed in c-kitPOS cells overexpressing PIM1, showing greater therapeutic efficacy and significantly reducing infarct scar [34].

Positive therapeutic effects have also been observed in the CDCs cell population [35–38]. Administration of CDCs to rats after an induced myocardial infarction contributed to the reduction of scar size and improved heart functionality. The improvement was maintained despite the evanescene of transplanted cells survival [36]. A positive effect was also observed in pigs [26, 38]. In large animal models, improvements in left ventricular function has been demonstrated [35, 39, 40] while maintaining left ventricular ejection fraction [41].

**Table 2:** Major cardiac stem cell research and preclinical studies

Study procedure	Animal model	Cells	Major findings	Date	Ref.
-isolation of c-kit^POS^ cells -immunocytochemistry analysis -induction of MI -implantation of myocytes cells	rat	c-kit^POS^ cells	-improved of functional performance of the postinfarcted hearts injected with Lin(-) c-kit^POS^ cells	2003	[25]
-using transgenic mice to determine the location of c-kit^POS^ cells in a healthy heart and in the heart after myocardial infarction	mouse	c-kit^POS^ cells	- the number of c-kit^POS^ cells is higher at birth compared to adults - an increased number of c-kit^POS^ cells was observed in the infarction region	2008	[32]
-isolation of c-kit^POS^ cells with high GATA-4 expression -co-culture of c-kit^POS^ GATA-4 cells with adult cardiomyocytes	rat	c-kit^POS^ cells	-c-kit^POS^ GATA-4 cells affect cardiomyocyte survival by inducing IGF1R	2010	[33]
-isolation of CDCs -induction of MI -intramyocardial injection of CDCs	pig	autologous CDCs	-preservation of left ventricular function - minimization of adverse ventricular remodeling	2011	[35]
-isolation of CDCs from rat and human hearts -induction of MI in rats -intramyocardially CDCc injection in groups: syngenic group, allogenic group, xenogeneic group	rat	allogenic CDCs syngenic CDCs	-allogenic CDCs promotes cardiac regeneration -improvement in cardiac function was observed in rat models	2012	[36]
-preparation of cardiospheres -injection cardiospheres in the peri-infarct zone	rat	allogenic CDCs	-reduced scar size -increased cardiac function	2013	[37]
-induction of chronic infarction -intramyocardial injection of CSCs	dog	autologous CSCs	-less increase in left ventricular end-systolic volume -preservation of left ventricular ejection fraction	2013	[41]
-induction of MI -intracoronary infusion of CDCs	pig	allogenic CDCs	-MRI is a useful tool for assessing dynamic changes in the infarct and monitoring regenerative efficacy -decreased scar size -increased myocardium viability	2013	[38]
-isolation and culture of human MSCs and CSCs -induction of MI in pig -intramyocardial injection of MSCS and CSCs	pig	Xenogeneic MSCs and CSCs	-reduced scar size -restoration of diastolic and systolic function of the left ventricle	2013	[39]
-development of the c-kit^POS^ cells isolation protocol	rat mouse	c-kit^POS^ cells	-identification and isolation of c-kit^POS^ cells	2014	[31]
-isolation of CSCs cells -intracoronary infusion of autologous CSCs	pig	autologous CSCs	-improves regional and global left ventricular function -promotes cardiac and vascular regeneration in pigs with old MI	2014	[40]
-preparation of CDCs -randomized -induction of MI -intracoronary administration of CDCs	pig	allogenic CDCs	-reduction of infract size -CDCs are effective in cardioprotection -prevention of microvascular obstruction	2015	[26]
-isolation of human CSCs -identification of c-kit^POS^ cells with PIM1 overexpression -intramyocardial injection of CSCs to Yorkshire swine	pig	human CSCs	-PIM1 overexpression enhanced the effect of intramyocardial delivery of CSCs to infarcted porcine hearts -reduced scar size	2017	[34]

CDCs - cardiosphere-derived stem cells, CSCs - Cardiac Stem Cells, MI - Myocardial Infarction, MRI - Magnetic Resonance Imaging, MSCs - Mesenchymal Stem Cells

After pre-clinical studies with MI models, which pointed out to improvement of left ventricular function and reconstitution of damaged tissues, first clinical trial SCIPIO was conducted (Table 3) [40, 42]. Despite promising results, the Lancet editors decided to retract the article [43]. Although recent studies which used genetically based lineage tracing of the cardiac c-kit^pos^ cell using Cre recombinase in mice proved that mostly give rise to ECs after myocardial injury [44–46]. On the other hand, advocates of c-kit^pos^ cells’ stemness emphasize that only a small fraction (~ 1–2%) of the c-kit^pos^ cell population shows multipotent characteristics, while the majority constitute of mast and endothelial/progenitor cells [47]. However, this only proves that c-kit alone should not be seen as a reliable biomarker of cardiac stem cells.

The second well described population is termed cardiosphere-derived cells (CDCs). These undifferentiated cells were first isolated from self-adherent clusters, termed cardiospheres, formed in the culture of cells from atrial or ventricular biopsy specimens. Such cells are heterogenous, express mesenchymal and progenitor cells’ markers, are clonogenic, capable of self-renewal and differentiation into muscle and vascular cells [48]. The Marbán group miniaturized and optimized this culture method such that only low amounts of starting material from minimally invasive percutaneous endomyocardial biopsies were required [49].

As results from the studies on MI models including mice, rat and pig seemed promising, clinical trial CADUCEUS which aimed at assessment of CDCs transplantation effectiveness in patients with acute MI was established. Phase I trial has demonstrated reduction of infarct size, increased viable heart mass and regional contractility, however there was no significant difference in left ventricular (LV) ejection fraction in the group which received autologous CDCs compared to control group, receiving standard medical treatment [50]. CADUCEUS clinical trials continued, particularly for ischemic heart disease, which were based on an autologous cell source [51]. The first clinical trial testing the administration of autologous c-kit(+) CSCs in patients with ischemic heart failure who underwent coronary artery bypass grafting was SCIPIO. The study described surgical procedures and also analyzed diagnostic imaging with cardiac MRI. Results indicated a reduction in scar tissue and an increase in myocardial regeneration [52]. Studies from the use of CSCs in pediatric patients demonstrate that intracoronary administration of the cells is feasible and safe, as well as providing therapeutic benefits. The clinical trial involved the administration of antologous, previously isolated cells [53]. Whereas the use of allogeneic cardiosphere material for myocardial regeneration was conducted in patients with ischemic disease and left ventricular dysfunction (ALLSTAR) [54]. The study was based on intracoronary administration of cells to qualified patients. ALLSTAR showed less cardiac remodeling after myocardial infarction and left ventricular regeneration, but without scar reduction. In turn, the PERSEUS clinical trial was based on the use of CDCs in the treatment of congenital heart failure. Intracoronary injection of autologous CDCs in patients with hypoplastic left heart syndrome was shown to have positive effects in reducing heart failure and favorable impacts on ventricular function [55, 56]. Studies on the possibility of regenerating the ischemic heart and left ventricular failure have been also conducted using human embryonic cardiac progenitor cells (ESCORT). Improved parameters were obtained, which were based on direct action but also on the paracrine effect of CPCs [57]. Prompt therapeutic intervention after an acute MI provides better conditions for myocardial regeneration, especially when scar formation has not developed so far. The CAREMI trial set out to evaluate the safety of administering allogeneic CSCs immediately after MI for regenerative purposes [58, 59]. An innovative study was conducted as a combination of transplantation of MSCs together with endomyocardium-derived antologous CSCs (CONCERT-HF) [60]. The positive effects of the combination therapy in patients with symptoms of myocardial ischemia were evaluated, and the safety of the therapy was determined. The administration of CSCs in the course of non-ischemic cardiomyopathies has been analyzed in subsequent studies (HOPE). Patients with Duchenne muscular dystrophy received allogeneic CPCs, resulting in decreased scar and improved ventricular systolic parameters [61]. Experimental studies using a porcine model of pediatric dilated cardiomyopathy were also recently conducted to analyze the potential of CDCs therapy and exosomal secretion mechanism (CDCex) [62]. The study demonstrated improved cardiac function and reduced fibrosis, which was mediated through exosomes containing proangiogenic and cardioprotective microRNAs.

**Table 3:** Clinical trials: the use of cardiac stem cells in cardiovascular disorders

NCT Number	Study name	Date	Phase	Patients	Cell source	Disease	Results	Ref.
NCT00893360	CADUCEUS	2009–2011	I	17	Autologous CDCs	ILVD	-increased viable of myocardium -decreased scar size -improved regional function of infarcted myocardium	[51]
NCT00474461	SCIPIO	2009–2013	I	33	Autologous CDCs	ICM	-reduction in infarct size -improving left ventricular function	[52]
NCT01273857	TICAP	2011–2013	I	14	Autologous CDCs	HLHS	-improvement of right ventricular ejection fraction	[53]
NCT01458405	ALLSTAR	2012–2019	I, II	134	Allogenic CDCs	ILVD	-not reduce scar size -decrease in left ventricular end-diastolic volume -decrease in left ventricular end-systolic volume	[54]
NCT01829750	PERSEUS	2013–2016	II	41	Autologous CDCs	HLHS	-reduced heart failure - favorable effect on ventricular function	[55, 56]
NCT02057900	ESCORT	2013–2018	I	6	CSCs	ILVD	-increased systolic motion of the cell-treated segments -patients with improved symptoms	[57]
NCT02439398	CAREMI	2014–2016	I, II	55	Allogenic CSCs	MI	-CSCs can be safely administered to patients after MI	[58, 59]
NCT02501811	CONCERT-HF	2015–2020	II	125	c-kit^POS^ cells and MSCs	IHD	-treatment is safe and feasible -the proportion of MACE was significantly decreased	[60]
NCT02485938	HOPE	2016–2017	I, II	25	Allogenic CDCs	CM - DMD	-decreased scar size -improvement of systolic thickening of the inferior wall	[61]
NCT03129568	TICAP-DCM	2017–2018	I	5	CDCs	DCM	-improved cardiac function	[62]

CDCs - Cardiosphere-Derived Stem Cells, CM - Cardiomyopathy, CSCs - Cardiac Stem Cells, DCM - Dilated Cardiomyopathy, DMD - Duchenne Muscular Dystrophy, HLHS - Hypoplastic Left Heart Syndrome, ICM - Ischemic Cardiomyopathy, IHD - Ischemic Heart Disease, ILVD - Ischemic Left Ventricular Dysfunction, MACE - Major Adverse Cardiac Events, MI - Myocardial Infarction, MSCs - Mesenchymal Stem Cells

Due to the small pool of CPCs and the difficulty of harvesting them, efforts are underway to obtain them from other sources [63], including iPSCs. iPSCs can provide an unlimited supply of cells and do not generate ethical problems (as in the case of embryonic cells). Through direct reprogramming and transdifferentiation of iPSCs, it is possible to rapidly obtain CPCs.

The possibility of using multipotent CPCs, which show multidirectional differentiation and, very importantly from a clinical point of view, have a lower oncogenic risk, qualifies these cells for clinical trials. Undifferentiated iPSCs, on the other hand, are associated with an oncogenic risk of uncontrolled growth. Attempts are being made to differentiate CPCs derived from iPSCs. However, obtaining such CPCs with characteristic surface markers without ex vivo genetic manipulation is becoming difficult. Meanwhile, any genetic manipulation poses the risk of uncontrolled growth after transplantation [64]. Unlike typical stem cells, pluripotent cells show limited differentiation abilities. They are usually more differentiated and are influenced by both their stem cells and the niche in which they develop. Concepts are therefore emerging to generate CPCs from human self-renewing pluripotent stem cells. However, challenging issues still arise. The first concerns the maturation of iPSC derived CPCs cells, as they do not reach stages more advanced than those in the fetal heart. In addition, these cells are difficult to target specific cardiomyocyte subtypes (atrial, ventricular). CPCs populations from iPSCs are also hard to maintain in a stable and pure state for long periods of time [65]. This includes limitations related to proliferation and the instability of the markers shown, confirming the state of the cells. Generating iPSCs-derived CPCs involves the use of different protocols, types of media and additives like growth factors. The first step involves the differentiation of cells into a pluripotent state using reprogramming factors for example: SOX2, OCT4, KFL4 and c-MYC [66]. Cardiac differentiation is necessary once the desired goal has been achieved. Various protocols are available, based on spheroid culture, as well as on a gel, monolayer culture with or without serum. The most commonly used is monolayer culture without serum (e.g., mTeSR1 or E8) [67, 68]. Frequently used culture reagents are: StemPro34, VEGF, DKK, DMEM/F12, B27, EGF, BPEL, PDGFα + PDGFβ, Wnt3A, bFGF, BMP4 [64, 69]– [71]. In culture, special attention should be paid to preserving pluripotency and cell self-renewal. The difficulty, however, is maintaining the homogeneity of such a culture. Activin-like kinase 5 inhibitors have also been described as novel and potent inducers of CPC differentiation into cardiomyocytes [72].

Despite the great potential for therapeutic use of CPCs derived from iPSCs, clinical solutions are not being obtained to date. However, these cells can be used in cardiac disease modeling, pharmacokinetic and genetic studies in search of signaling pathways important for cardiomyocyte differentiation [73].

## Paracrine mechanism of CPCs’ action as the part of driving force of damaged myocardium regeneration

**Fig. 1:**
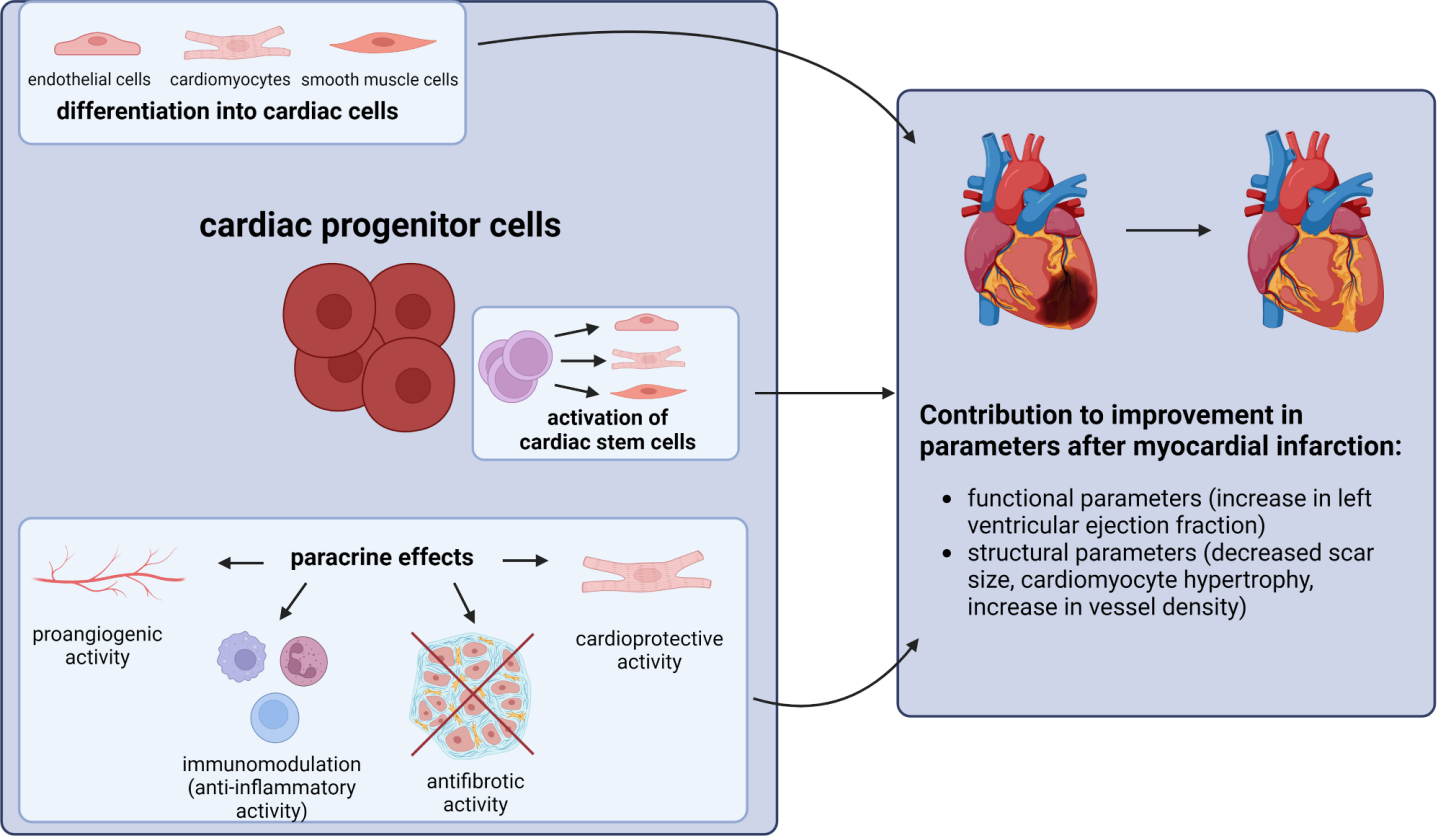
General routes of influence of cardiac progenitor cells on heart regeneration after myocardial infarction. Cardiac progenitor cells may act via three main mechanisms to contribute to cardiac repair after damage. They include: direct differentiation into cardiac cells such as cardiomyocytes, endothelial cells and vascular smooth muscle cells; activation of cardiac stem cells to differentiate into different cardiac cells; and paracrine effects by which CPCs promote angiogenesis and cardioprotection while suppressing fibrosis and inflammation. Preclinical and clinical studies show that transplantation of cardiac progenitor cells and administration of their acellular products lead to improvement in functional and structural parameters of the heart after myocardial damage (Created with BioRender.com)

Results of experimental studies on cardiac regeneration clearly show that the number of cardiomyocytes differentiating form cardiac progenitor cells transplanted at the site of injury is too small to explain the observed improvement in heart function [74]. It has been therefore suggested that the paracrine release of factors which have a positive effect on damaged myocardium is an important mechanism of CPCs’ action (Fig. 1) [74–76].

## Cardiac progenitors’ secretome facilitates myocardial regeneration after injury

**Fig. 2:**
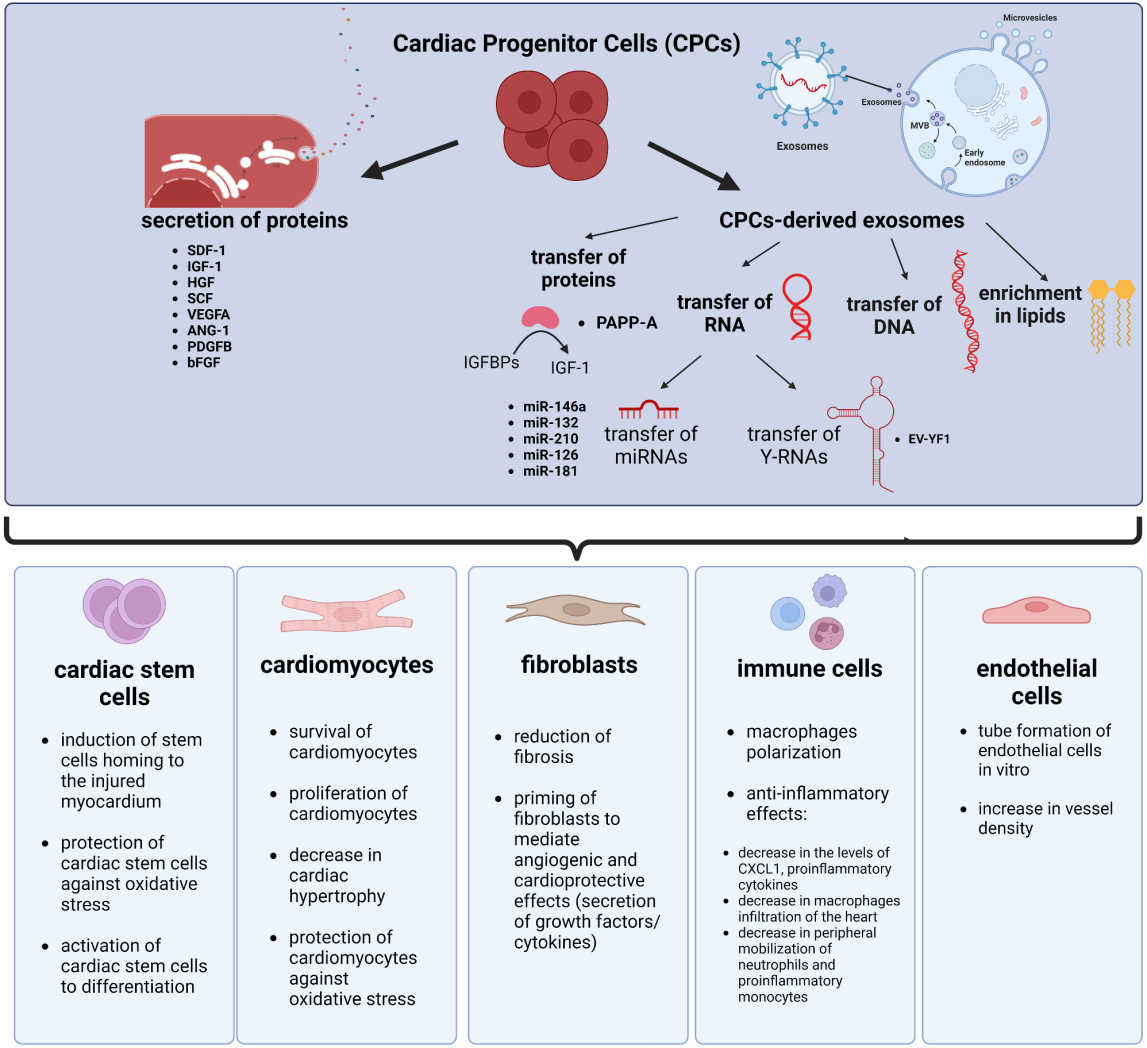
Paracrine mechanisms of action of cardiac progenitor cells on heart-derived cells. Cardiac progenitor cells exert paracrine effects on cardiac stem cells, cardiomyocytes, cardiac fibroblasts, immune cells and endothelial cells contributing to repair of myocardium after injury. Two main characterized routes of paracrine action include secretion of proteins or release of exosomes rich in protein, DNA, RNA and lipid cargoes. Abbreviations: ANG-1 - Angiopoietin 1, bFGF - Basic Fibroblast Growth Factor, HGF - Hepatocyte Growth Factor, IGF-1 - Insulin-like Growth Factor 1, IGFBPs – Insulin-like Growth Factor Binding Proteins, PAPP-A - pregnancy-associated plasma protein-A, SCF - Stem Cell Factor, SDF-1α - Stromal cell Derived Factor 1α, VEGFA - Vascular Endothelial Growth Factor A, PDGFB - Platelet Derived Growth Factor subunit B (Created with BioRender.com)

CPCs may produce and secrete a variety of growth factors, cytokines and chemokines (Fig. 2) [77]. Several paracrine factors released by adult cardiac progenitor cells, important players in reparation of myocardium after injury, have been characterized. Their effects range from inhibition of cardiomyocytes apoptosis, promotion of angiogenesis to promotion of function, recruitment, and proliferation of stem cells. They include: HGF (Hepatocyte Growth Factor), IGF-1 (Insulin-like Growth Factor 1), SCF (Stem Cell Factor) and SDF-1α (Stromal cell Derived Factor 1α), and ANG-1 (Angiopoietin 1), VEGFA (Vascular Endothelial Growth Factor A), PDGFB (Platelet Derived Growth Factor subunit B) and bFGF (Basic Fibroblast Growth Factor) [78]. A positive influence of transplantation of exogenous CDCs on endogenous cardioblast activation in injured hearts has been described. Functional studies demonstrated that SDF1, secreted by CDCs, was a crucial factor inducing increase in replenishment of lost cardiomyocytes [79]. It has been also shown that injection of CPCs, namely cardiosphere-derived cells, especially those derived from patients diagnosed with heart failure led to increase in left ventricular ejection fraction, thickest infarct wall and lesser scarring in mouse model of MI and that, as it has been revealed may be attributed to secretion of SDF-1, a pro-angiogenic and cardioprotective factor [80]. Interestingly, explant-derived cardiac cells overexpressing SDF-1α, which promotes angiogenesis and stem cell recruitment, were also generated. Transplantation of SDF-1α overexpressing cardiac stem cells in the mouse model of MI resulted in enhancement of the cardiac function, promoted angiogenesis, recruitment of bone marrow cells and generation of new cardiomyocytes, reduced scarring, and myocytes apoptosis [81].

IL-6 released by cardiac progenitor cells in large amounts, has also a documented role in reparation of injured myocardium. A study for 2017 points out to the role of this cytokine in promotion of cardiac reparation, macrophages polarization and proliferation of cardiomyocytes, as well as reduction of fibrosis [82]. Toran et al. described a pro-angiogenic activity of chemokine CXCL6 by CXCR2 receptor. This chemokine is released in larger amounts by cardiac progenitor cells when compared to secretome of human dermal fibroblasts or mesenchymal stem cells [83]. Transplantation of explant-derived cardiac stem cells, which overexpress IGF-1, enhanced the long-term engraftment in a mouse model of MI and improved myocardial repair. In addition, IGF-1 overexpression promotes EDCs and cardiomyocytes viability [84].

## CPCs exosomes contain non-coding RNAs (ncRNAs) and proteins with cardioprotective functions

Although as it was mentioned in the previous chapter the mechanism of positive influence of cardiac progenitor cells is indirect and CPCs can secrete various cytokines, chemokines and factors mediating these effects, CPCs-derived exosomes and their cargo has also an important contribution to the paracrine mechanism of their action (Fig. 2).

Exosomes (30–150 nm in diameter) are extracellular vesicles (EVs) of endosomal origin, key mediators of intercellular communication [85]. As cell-free structures, exosomes are expected to ensure the safety of the applied therapy, and the possibility of using them as carriers undeniably offers many opportunities. The use of extracellular vesicles in cardiac tissue repair is undoubtedly a type of next-generation therapy [86]. The trophic effects obtained after using conditioned medium from CPCs culture prove that exosomes are an active component in myocardial regenerative therapy.

Extracellular vesicles are a heterogeneous group of spherical structures, composed of a lipid bilayer and express on their surface antigens specific to their parent cells. They are an indispensable link in intercellular communication due to the fact that they present a variety of active substances on their surface. They act as carriers, transporting proteins, lipids and nucleic acids, and once they have reached another cell, they can regulate their gene expression [87] In addition to the rich composition of the internal content of exosomes, they carry mRNA molecules, which can be translated into proteins after entering the target cell, indicating the important role of exosomes as vectors of genetic information. Exosomes have been shown to be secreted from cardiac telocytes in areas affected by myocardial infarction, indicating their potential role in tissue regeneration through angiogenesis [88].

The presence of exosomes derived from CPCs was demonstrated by electron microscopy images of the ultrastructure of mouse and human CPCs [87]. The diameter of EVs emitted by CPCs is about 30-90 nm [89]. Studies on animal models of MI confirm that administration of CPC-derived exosomes mimics the effects observed when cardiac progenitor cells are transplanted. First studies were done in mice model of MI where it has been shown that administration of exosomes can mimic the benefits observed in case of CDC transplantation [90]. The positive influence of CPC-derived exosomes has been confirmed not only in small but also large animal model of the disease, namely porcine where it has been shown that intramyocardial administration of these nanovesicles decreased infarct size and preserved left ventricular ejection fraction (LVEF). Additionally, application of CDC-derived exosomes led to decrease in cardiomyocyte hypertrophy and left ventricle collagen content and an increase in vessel density [91].

**Fig. 3:**
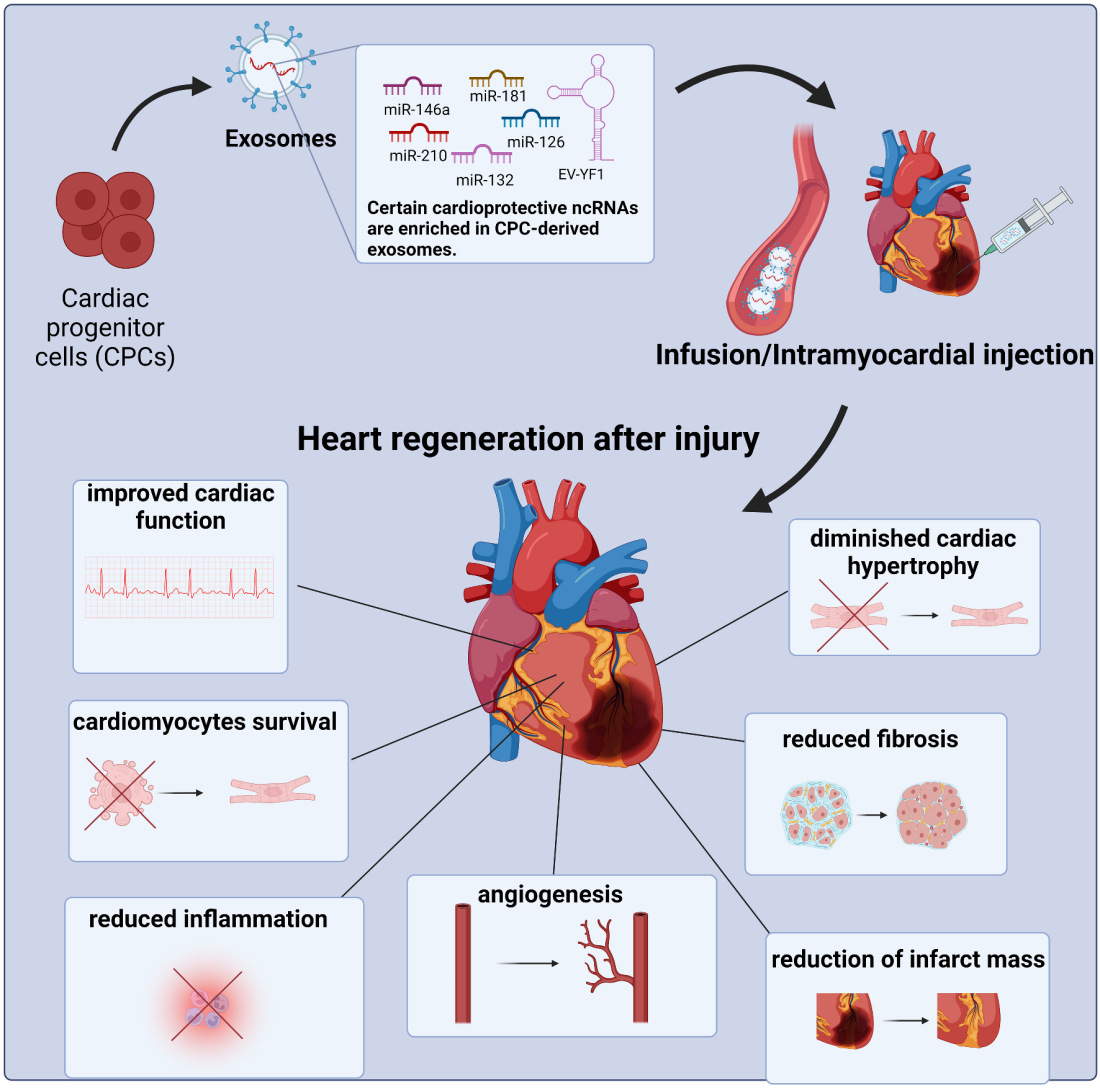
Non-coding RNAs enriched in cardiac progenitor cell-derived exosomes promote heart regeneration after injury. CPC-derived exosomes which are rich in cardioprotective non-coding RNAs, including miRNAs and YRNAs, can be administered via intramyocardial injection or intracoronary infusion to the damaged heart and exert beneficial effects including reduction of fibrosis and infarct mass, reduced inflammation and cardiac hypertrophy and promotion of cardiomyocytes survival, angiogenesis and improvement of cardiac function Abbreviations: CPCs – cardiac progenitor cells, ncRNAs – non-coding RNAs (Created with BioRender.com)

The positive influence of cardiac progenitor cells’ exosomes on injured heart is however not limited to cardiomyocytes, it has also been reported for other cell types present in the heart. Exosomes and other extracellular vesicles derived from human cardiosphere cells have been shown to prime dermal fibroblasts to mediate angiogenic and cardioprotective effects, namely reduction of scar mass, increase in global pump function and vessel density in rat MI model. Primed fibroblasts secreted larger amounts of the abovementioned SDF-1 and VEGF as well as shown differential expression of miRNAs when compared to unprimed fibroblasts and cardiosphere-derived cells [92]. Exosomes can carry various cargo which dictates their role in recipient cells – proteins, metabolites, lipids as well as nucleic acids including non-coding RNAs influencing gene expression [93–95]. It has been suggested that CPCs’s exosomes have stronger cardioprotective and proangiogenic activity and lead to improvement of LVEF, reduced scarring, increased blood vessel density in the infarct region in a study that compared the influence of administration of cardiac-resident progenitor cells (CPCs) and bone marrow-derived mesenchymal stem/progenitor cells (BMCs)-derived exosomes on cardiomyocyte apoptosis, tube formation by endothelial cells in vitro and on regeneration after myocardial ischemia in a rat model. The mediation of cardioprotective effects has been attributed, at least partially, to pregnancy-associated plasma protein-A (PAPP-A). PAPP-A, the most upregulated protein in exosomes of CPC vs. BMC, expressed on the surface of these vesicles leads to proteolysis of IGFBPs to IGF-1, which in turn leads to activation of IGF-1 receptor, Akt and ERK1/2 phosphorylation, decreased caspase-7 activation and prevention of cardiomyocytes apoptosis [96]. An increasing body of research has also reported an important role of non-coding RNAs in promoting damaged myocardium reparation (Fig. 3).

microRNAs (miRNAs) are short non-coding RNAs, approximately 22 nucleotides in length which may regulate even 60% of all protein-coding genes in mammals [97, 98]. This is the most frequently and probably the longest studied ncRNAs class, nevertheless many issues connected to their biogenesis and mechanisms of gene expression regulation remain unresolved [99, 100]. It is commonly recognized that depending on the level of complementarity between miRNA and target mRNA, the process of silencing of gene expression can take two different turns. miRNAs usually interact with the 3′ untranslated region (3′ UTR) of mRNA subsequently leading to repression of translation, mRNA deadenylation and decapping. If there is perfect complementarity between the two interacting RNA molecules, mRNA gets degraded [101]. miRNAs are crucial regulators of heart development, physiological processes, including function of cardiomyocytes and other cell types of the heart and play role in disease states, including cardiovascular diseases [102–104].

Already in 2014 Ibrahim et al. point out that exosomes are mediators of indirect effects of CDCs promoting angiogenesis as well as proliferation and viability of cardiomyocytes. CDCs’ exosomes mimicked the activity of the cells themselves as after administration of these EVs in mouse MI model, improvement of both functional and structural parameters was observed, similarly as in case of CDCs’ transplantation. Inhibition of exosomes production blocked CDC-mediated benefits. What was emphasized in this study was the role of exosomal miRNAs, especially enrichment of miR-146a in CDC-derived exosomes as compared to NHDF (normal human dermal fibroblasts)-derived exosomes and in case of administration of these exosomes to post-MI hearts. Furthermore, in case of knockout of this miRNA, impaired heart function was observed. Treatment of mouse model of chronic MI with miR-146a mimic led to increased viable tissue, thicker infarcted walls and less adverse remodeling, reproducing some of the CDC-derived exosomes mediated benefits [90]. Explant-derived cardiac progenitor cells’ media is important for survival of cardiomyocytic cells and promotes tube formation in endothelial cells. Depletion of exosomes from the media abrogated these effects. They also point out two miRNAs which were enriched within CPC-derived exosomes as compared to NDHF-derived exosomes - an anti-apoptotic miR-210 and a pro-angiogenic miR-132 which downregulated their known targets. Administration of CPC-derived exosomes to infarcted rat hearts led to improved LV ejection fraction and structural benefits, including reduced scarring, enhanced angiogenesis and reduces cardiomyocyte apoptosis [89].

Furthermore, potential influence of exosomes isolated form rat CPC which were subjected to either normoxic or hypoxic conditions on heart cells. It has been demonstrated that hypoxic CPC-derived exosomes enhanced tube formation of cardiac endothelial cells and caused decrease of CTGF, COLIII and VIM mRNAs in cardiac fibroblasts treated with TGF-β. Interestingly, sonication of exosomes as well as treatment with inhibitor of RNA-induced silencing complex, consisting of effector proteins involved in gene expression silencing by miRNAs, led to diminishment of proangiogenic effects of EVs. This may suggest potential role of exosomal miRNAs in this process. Under that reasoning 11 miRNAs upregulated in hypoxic CPC-derived exosomes as compared to normoxic CPC-derived exosomes have been identified of which 7 were verified as truly differentially expressed by RT-qPCR. Next, to characterize covarying relationships between differential expression of miRNAs and conditions to which CPCs were subjected (normoxic or hypoxic) principal component analysis was applied. It pointed out 4 distinct clusters of covarying miRNAs. For further exploration of cue–signal–response relationships understood by relationships of these miRNA clusters levels, oxygen treatment of CPC and the biological response (tube formation or CTFG expression as fibrosis marker), Gray et al. applied modelling using the partial least square regression analysis. Finally, pro-regenerative influence of hypoxic CPC-derived exosomes in vivo was confirmed, as their administration significantly reduced fractional shortening of the left ventricle and reduced fibrosis in rat infarcted hearts [105].

Exosomal miRNA transfer can also be crucial for polarization of cardiac macrophages. Acute myocardial infarction triggers innate immune response, in which neutrophiles activation followed by monocytes/macrophages activation occurs. Importantly, the process of infiltration of monocytes/macrophages is crucial for the infarct size. Exosomes are the element of CDCs’ secretome, which mimics cardioprotective effects of CDCs in rat and pig model of MI. Treatment of macrophages isolated from MI-hearts with CDC-derived exosomes led to a distinctive shift in macrophages (Mϕ) polarization. Exosomes from CDCs and fibroblasts were sequenced and interestingly, highest changes in expression were observed for miRNAs. 2 miRNAs have shown significantly deregulated expression in CDC-derived exosomes (CDC_exo_), namely miR-126 and miR-181. Curiously, miR-181a/b had differentially expressed target genes in CDC_exo_-treated Mϕ. RNA sequencing of CDC_exo_-primed rat bone marrow-derived Mϕ also pointed out miR-181a/b as the most highly upregulated miRNA implying it as a possible important regulatory exosomes’ cargo. Functional studies have shown a significant influence of miR-181b on reduction of infarct size and Mϕ infiltration and it was suggested that inhibition of PKC- δ, a regulator of inflammation, by miR-181b in CDC_exo_ potentially underlined the cardioprotection induced by CDCs [106].

YRNAs are another class of short (less than 100 nucleotides) non-coding RNAs with specific secondary structure. Human has four YRNA genes [68, 107]. First described in 1981 in complexes with La and Ro60 proteins, YRNAs harbor many protein binding sites, due to which they can dictate RNA-binding proteins’ transport, splicing, ncRNAs quality control and processing [107–109]. By participating in formation of replication forks, YRNAs are also associated with DNA replication regulation [110]. Furthermore, YRNAs can be processed into shorter fragments, the so called YRNA-derived fragments which can be released during apoptosis, innate immune system activation and are potentially involved in gene regulation [111].

High enrichment of YRNAs appear in CDC-derived exosomes (18% of all ncRNAs), and the YRNA appeared to have protective effects on H_2_O_2_-induced oxidative stress in neonatal rat ventricular myocytes [112].

YRNAs present in CDCs’ exosomes have been characterized more extensively emphasizing EV-YF1, the most abundant RNA in CDC-derived exosomes [113]. Abundance of EV-YF1 correlated with CDCs’ potency, understood as increased post-MI ejection fraction after intramyocardial injection compared to placebo. EV-YF1 seems to be packed to EVs specifically by CDCs and transferred to the cytoplasm of bone marrow-derived macrophages, where it recapitulates some of the effects mediated by CDCs’ exosomes - EV-YF1 induces increase in *Il10* gene expression as well as IL-10 protein secretion. This increase in secretion of IL-10 from EV-YF1-primed BMDMs leads to protection of cardiomyocytes from oxidative stress in I/R (ischemia-reperfusion) in vitro model. Further studies in vivo suggest a decrease in infarct mass, in the number of CD68^+^ macrophages within the infarct as well as apoptotic cardiomyocytes [113].

EV-YF1 in exosomes was also an object of study in the field of hypertension. In an in vitro and in vivo model of cardiac hypertrophy induced by chronic infusion of Ang (angiotensin) II both the ncRNA as well as the exosomes diminished cardiac hypertrophy and fibrosis and had an anti-inflammatory effect. In a model of Ang II–induced kidney injury, EV-YF1 and CDC_exo_ led to improvement of kidney function and decrease in renal inflammation and fibrosis. EV-YF1 and CDC_exo_ prevented angiotensin II–induced end-organ damage by modulating IL-10 secretion [114].

After demonstrating the important function of EV-YF1 in MI and hypertension, further investigation was focused on hypertrophic cardiomyopathy. In mouse transgenic model of this disease with a relevant mutation (cTnI^Gly146^), it has been shown that EV-YF1 inhibits cardiomyocyte hypertrophy and fibrosis and does that by immunological modulation and alteration of macrophages transcriptomic profile. More specifically, treatment with EV-YF1 decreases interstitial LV fibrosis and cardiomyocyte hypertrophy and downregulates JNK and Smad pathways associated with these processes. It also reduced CXCL1 expression in cardiomyocytes, proinflammatory cytokine expression, macrophage infiltration of the heart as well as peripheral mobilization of neutrophils and proinflammatory monocytes. Infusion of EV-YF1 improved mobility of the mice and their cardiac function. All this data implies that EV-YF1 may be a promising therapeutic agent for hypertrophic cardiomyopathy [115].

## Concluding remarks and future perspectives

In the past 15 years interest in cardiac progenitor cell therapy as a potential novel pro-regenerative strategy of heart failure treatment has risen. Cardiac progenitor cell transplantation has reached clinical testing and multiple cell types have been proposed to exert beneficial effects including cardiosphere-derived cells, and CD117^+^ cardiac stem cells [42, 50]. It has been also increasingly understood that the primary mechanism of action of cell therapy is not based on and limited to direct differentiation into cardiomyocytes but is rather based on paracrine signaling and the positive influence on heart include angiogenesis, cardioprotection, and anti-fibrotic activity. Cardiac progenitor cells can secrete cytokines, chemokines, and growth factors as well as exosomes, rich in protein, lipids and nucleic acids which can all contribute to the positive effects. Exosomes themselves can mimic the benefits of the transplanted cells making them a promising alternative to cell-based therapy. By functioning as a carrier and having a biologically active payload, exosomes may exert a multitude of potential effects and become an attractive therapeutic tool [116]. Importantly, they are also selectively taken up by specific recipient cells. Exosomes are potentially immune privileged. They can also be stored long-term making them more suited as a therapeutic agent [117]. Nevertheless, there are several limitations to the use of exosomes. Firstly, the mechanism of cargo packaging remains unknown. The content of the exosomes may depend on the state of the donor cells so standardization of conditions in which exosomes are collected should be implemented [118]. Exosomes have also a short half-life which forces repeated injections for prolonged effect [119]. Their delivery by infusion to the injured heart still poses a challenge as has been shown in a study evaluating 2 routes of delivery – IM (intramyocardial) delivery and IC (intracoronary) infusion, proving IC to be ineffective as compared to donor cells [91]. Heart is also an organ comprising of a multitude of cell types, including cardiomyocytes, fibroblasts, endothelial cells, stem cells and interstitial cells and influence of exosomes on each of these cell types should be carefully studied before implementing any therapy [117]. Another limitation is the knowledge on cargo biology. Although microRNAs are studied in mammals for two decades now, many issues related to their biogenesis and activity remain unresolved. Looking from a therapeutic angle, the specificity, dosing, and the feature of targeting multiple mRNAs and competition for binding of a particular target may be some of the issues which have to be addressed [120]. YRNAs, although potentially promising as exemplified by EV-YF1, are also not that extensively studied and basic research on their biology will be necessary for deeper understanding of their action.

Numerous ongoing preclinical and clinical studies are examining the feasibility of using stem cell therapy for cardiovascular diseases. The growing number of patients and heart transplantation, which is often the only possible solution to end-stage heart failure, is prompting the development of other treatments. For years, therapies based on regenerative medicine have been creating new possibilities and perspectives. Advanced research and experiments are directed at selecting a population of cells that would be established in the myocardium and differentiate into functioning cardiomyocytes. However, for various reasons, achieving this goal has not been satisfactory to date. One reason for the ineffectiveness of the therapy may be the high loss of transplanted cells, reaching more than 90%, which may be related to the lack of adhesion of these cells to the changed extracellular environment of the pathologically altered heart tissue [121].

Despite the promising results obtained in clinical trials, most often there is no physical evidence confirming the mechanism of action of a given therapy, and the effects obtained in trials are attributed to paracrine activities. In addition, it is worth mentioning that the lack of a blinded trial (control) makes it difficult to assess the achieved treatment effects. The question of the cell administration itself is not insignificant. During open chest procedures, the cells are administered directly to the heart, but this involves a high degree of invasiveness. In contrast, the administration of cells by injection into the coronary vessels is safer, although it may result in a smaller pool of cells that will finally reach the target site.

People suffering from cardiovascular disease are generally elderly persons who additionally have other metabolic conditions. The pool of their CPCs in the heart is greatly reduced and their regenerative capacity is diminished. It is also unknown how continuously taken medications affect the activity of CPCs. Unquestionably, discoveries related to the regenerative capacity of the myocardium have brought new research directions and possibilities for future therapies, for currently incurable diseases. With the numerous discoveries in this field, it is clear that methods must be developed to optimize the acquisition, reprogramming and maintenance of stable populations of CPCs that can be used in therapies. Developments in bioengineering sciences are creating additional opportunities for the application of these cells using biomaterials based on tissue engineering, but also genetic engineering (in connection with iPSCs).

## Data Availability

Not applicable.

## Abbreviations

HF: heart failure
TAH: total artificial heart
CPC: cardiac progenitor cell
EC: endothelial cell
CDC: cardiosphere–derived cell
MI: myocardial infarction
cCFU: Fs–cardiac colony–forming unit fibroblasts
CSPCs: cardiac side population cells
CSCs: cardiac stem cells
EPDCs: Epicardium–derived progenitor cells
HGF: Hepatocyte Growth Factor
IGF: 1–Insulin–like Growth Factor 1
SCF: Stem Cell Factor
SDF: 1α–Stromal cell Derived Factor 1α
ANG: 1–Angiopoietin 1
VEGFA: Vascular Endothelial Growth Factor A
PDGFB: Platelet Derived Growth Factor subunit B
bFGF: Basic Fibroblast Growth Factor
EDCs: explant–derived cardiac stem cells
EVs: extracellular vesicles
LVEF: left ventricular ejection fraction
BMCs: bone marrow–derived mesenchymal stem/progenitor cells
CDCexo: CDC–derived exosomes
I/R: ischemia–reperfusion
